# Singlet–Triplet Inversions in Through-Bond Charge-Transfer States

**DOI:** 10.1021/acs.jpclett.4c02317

**Published:** 2024-09-26

**Authors:** J. Terence Blaskovits, Clémence Corminboeuf, Marc H. Garner

**Affiliations:** Laboratory for Computational Molecular Design, Institute of Chemical Sciences and Engineering, École Polytechnique Fédérale de Lausanne (EPFL), 1015 Lausanne, Switzerland

## Abstract

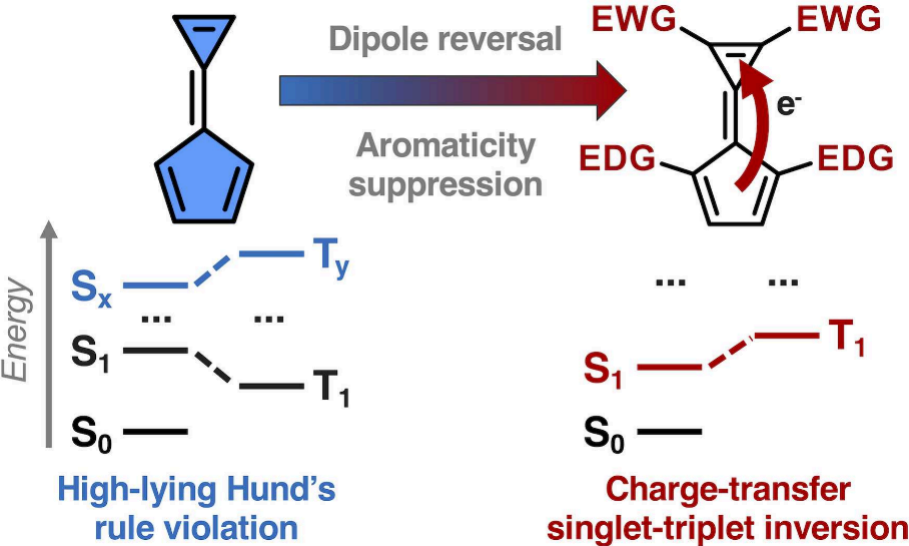

Molecules where the lowest excited singlet state is lower in energy than the lowest triplet are highly promising for a number of organic materials applications as efficiency limitations stemming from spin statistics are overcome. All molecules known to possess such singlet–triplet inversions exhibit a pattern of spatially alternating but nonoverlapping HOMO and LUMO orbitals, meaning the lowest excited states are of a local character. Here, we demonstrate that derivatives of the bicyclic hydrocarbon calicene exhibit Hund’s rule violations in charge-transfer (CT) states between its rings. These CT states can be tuned with substituents, so that the first excited singlet and triplet state are energetically inverted. This provides a conceptual connection between the emerging fields of inverted gap molecules and existing molecular design rules for state-of-the-art thermally activated delayed fluorescence materials.

Molecules in which the first excited singlet state (S_1_) is lower in energy than the lowest triplet state (T_1_) are poised to make significant contributions to the field of organic electronic materials. Such a class of inverted singlet–triplet gap materials may overcome existing limitations stemming from spin statistics in, among other applications, organic light-emitting diodes (OLEDs), photocatalysts, and lasers.^1^ Inverted gap molecules are a specific case of Hund’s rule of maximum spin multiplicity being violated in the lowest excited states (red in Figure 1a).^2^ While such inversion is not the only condition an emitter must have,^3−5^ a heptazine-based OLED device provided proof-of-concept for a new generation of molecules with the potential for achieving thermally activated delayed fluorescence (TADF) behavior.^6−9^ However, violations of Hund’s rule can exist among higher excited states (blue in Figure 1a), although such cases will not affect the photophysical rates in an emitter. At this point, two types of compounds with inverted singlet–triplet gaps have been classified, namely azaphenalenes^6−22^ and nonalternant polycyclic hydrocarbons^23−33^ (Figure 1b).

**Figure 1:**
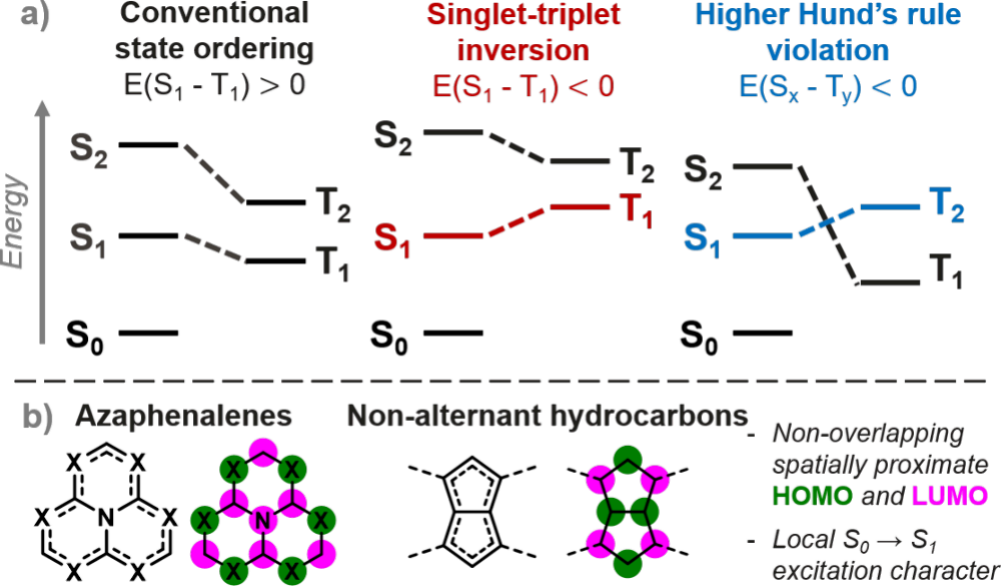
(a) Schematic of three situations in the ordering of excited states: all considered states respect Hund’s rule (black); Hund’s rule is violated in the lowest excited singlet and triplet states (red); Hund’s rule is violated in states other than the lowest excited states (blue). (b) The ring motifs underpinning existing azaphenalenes and nonalternant hydrocarbons with negative singlet–triplet gaps.

All reported compounds with Hund’s rule violations conform to a mechanism by which the frontier molecular orbital (MO) features are located on alternating atoms in a proximate but nonoverlapping (disjoint) fashion (Figure 1b).^34^ As such, the relevant excitations are delocalized throughout the molecule and do not exhibit electron–hole separation. This includes azaphenalenes^12,13^ and nonalternant pentalenic molecules,^23−25,27−29^ as well as larger multiresonant TADF emitters which have small positive singlet–triplet gaps.^35−37^ We recently explored the Hund’s rule violations in isopyrene, a pentalenic dimer of azulene, and *trans*-bicalicene, a dimer of calicene, see Figure 2a,b.^27^ Azulene is well-studied for its charge-separated ground state, large dipole moment and anti-Kasha emission.^38−40^ We and others recently reported substituted azulene derivatives that achieve excited-state Hund’s rule violations.^29,32^

**Figure 2:**
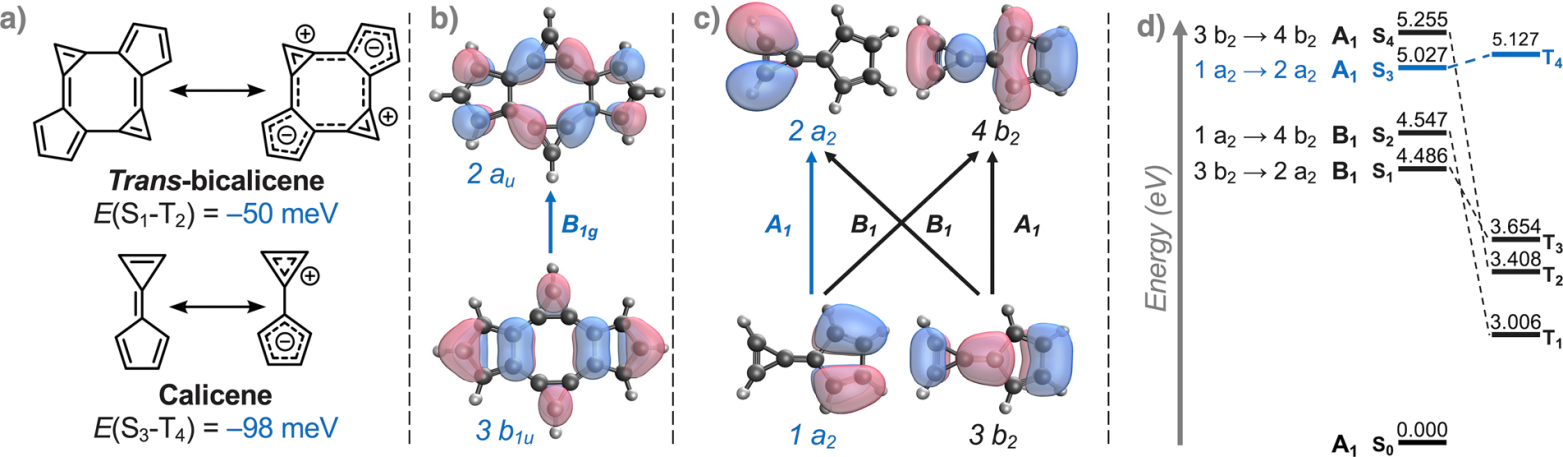
(a) The ground-state push–pull character in *trans*-bicalicene and calicene. (b) The nonoverlapping HOMO and LUMO contributing to the Hund’s rule violation in *trans*-bicalicene. (c) The quasi-degenerates HOMOs (below) and LUMOs (above) of calicene and the configurations that dominate its first four singlet and triplet excited states. (d) The first four singlet and triplet vertical excited state energies of calicene. The transition and orbitals that correspond to the Hund’s rule violation are indicated in blue in b–d.

Small singlet–triplet gaps are also achieved in donor–acceptor-type TADF emitters, where charge transfer (CT) involves MOs on spatially separate fragments. Small MO overlaps–and by extension a small exchange integral–are a requirement for an inverted gap.^13^ Still, such molecules have hitherto only been reported with small but positive gaps, which highlights the challenge in finding molecules with negative gaps, despite the ease of designing a molecule with minimal frontier MO overlap.

Calicene (triapentafulvalene) formally consists of cyclopentadiene (five-membered) and cyclopropylene (three-membered) rings linked by an exocyclic double bond. As with azulene, the ground-state structure of calicene is aromatically stabilized owing to the push–pull effect between the rings (Figure 2a).^41−44^ However, while azulene respects Hund’s rule in its excited states,^29,38^ calicene does not. In this letter, we explore the potential for controlling Hund’s rule violations in calicene derivatives. Through analysis of the charge-transfer (CT) mechanism of its excited states, we show that the frontier MO energetics can be systematically tuned to achieve inversion of the S_1_ and T_1_ states.

We evaluate the excited state gaps by computing the vertical excitation energies using equations-of-motion coupled cluster with singles and doubles (EOM-CCSD)^45^ and the cc-pVDZ basis. Structures are optimized in the ground state at the ωB97X-D/def2-TZVP level and are identified as energy minima with no imaginary frequencies (see Supporting Information for full computational details). EOM-CCSD was chosen as it provides an accurate and slightly conservative assessment of excited-state singlet–triplet gaps in organic molecules,^46,47^ and we compare with several other approximated CC methods with larger basis sets in Table S1 of the Supporting Information. Furthermore, our results for calicene are consistent with previous reports at the CASPT2 level, where a Hund’s rule violation was located, albeit between the S_1_ and T_2_ states.^42^

A closer look at the frontier orbitals of calicene reveals two quasi-degenerate highest occupied and lowest unoccupied MOs (HOMOs and LUMOs, Figure 2c). The first four singlet and triplet excited states of calicene are each described predominantly by a single-particle transition between these four possible MO pairs (Figure 2c-d). The states are ordered differently in the singlet and triplet manifolds due to varying small contributions from other electron configurations. The states are paired in Figure 2d based on the weights of the MO configurations and those of the natural transition orbitals (NTOs, see Figure S1). This analysis indicates that a Hund’s rule violation exists between the S_3_ and T_4_ states, although we note that the magnitudes of the MO/NTO amplitudes vary somewhat between these states. A robustly negative energy gap of *E*(S_3_–T_4_) = −98 meV between these states is observed at the EOM-CCSD/cc-pVDZ level (Figure 2d).

The S_3_-T_4_ state pair exhibiting the Hund’s rule violation involves a nonoverlapping HOMO and LUMO of *a*_2_ symmetry localized on the cyclopentadiene and cyclopropylene fragments, respectively (blue in Figure 2c-d). These states thus constitute a charge-transfer (CT) state with 95% CT character from the 5-membered to the 3-membered ring in S_3_, and a separation of the electron and hole of 3.6 Å (see Supporting Information). This differs qualitatively from the Hund’s rule violation in *trans*-bicalicene, in which the contributing orbitals are both located simultaneously on the 3- and 5-membered rings in an alternating fashion (Figure 2b). This corresponds to only 37% inter-ring CT character in S_1_ and a net electron–hole separation of 0.0 Å, indicative of locally excited states. The other three excited state pairs in calicene, which adhere to Hund’s rule, involve two other π-type orbitals of *b*_2_ symmetry (black in Figure 2c). These orbitals are delocalized over the entire molecule, and therefore exhibit significant overlap with each other and with the *a*_2_ orbitals, leading to preservation of Hund’s rule in these three singlet–triplet state pairs.

While a violation of Hund’s rule in the excited states is of theoretical interest, it is not optically relevant for the design of optoelectronic materials. Therefore, by stabilizing the LUMO localized on the cyclopropylene fragment and destabilizing the HOMO on the cyclopentadiene relative to the delocalized *b*_2_ orbitals, we show that the energies of the singlet and triplet states violating Hund’s rule can be lowered relative to the three other excited states.

It is well-known that the substitution of conjugated systems with electron-withdrawing (-donating) groups stabilizes (destabilizes) their frontier MOs.^48,49^ Upon substituting the 3-membered ring with a strongly electron-withdrawing *pull* moiety (−CN, magenta in Figure 3), the energies of all four FMOs shown in Figure 2c are stabilized. The *a*_2_ LUMO, which is localized on the 3-membered ring, is the most strongly impacted, with its energy lowered by more than 3 eV, while smaller energy lowerings are observed on the other orbitals. As a result of this stabilizing effect on the CT-type LUMO, the Hund’s rule inversion is preserved but is lowered from S_3_ to S_2_ with *E*(S_2_–T_4_) = −28 meV, Figure 3.

**Figure 3:**
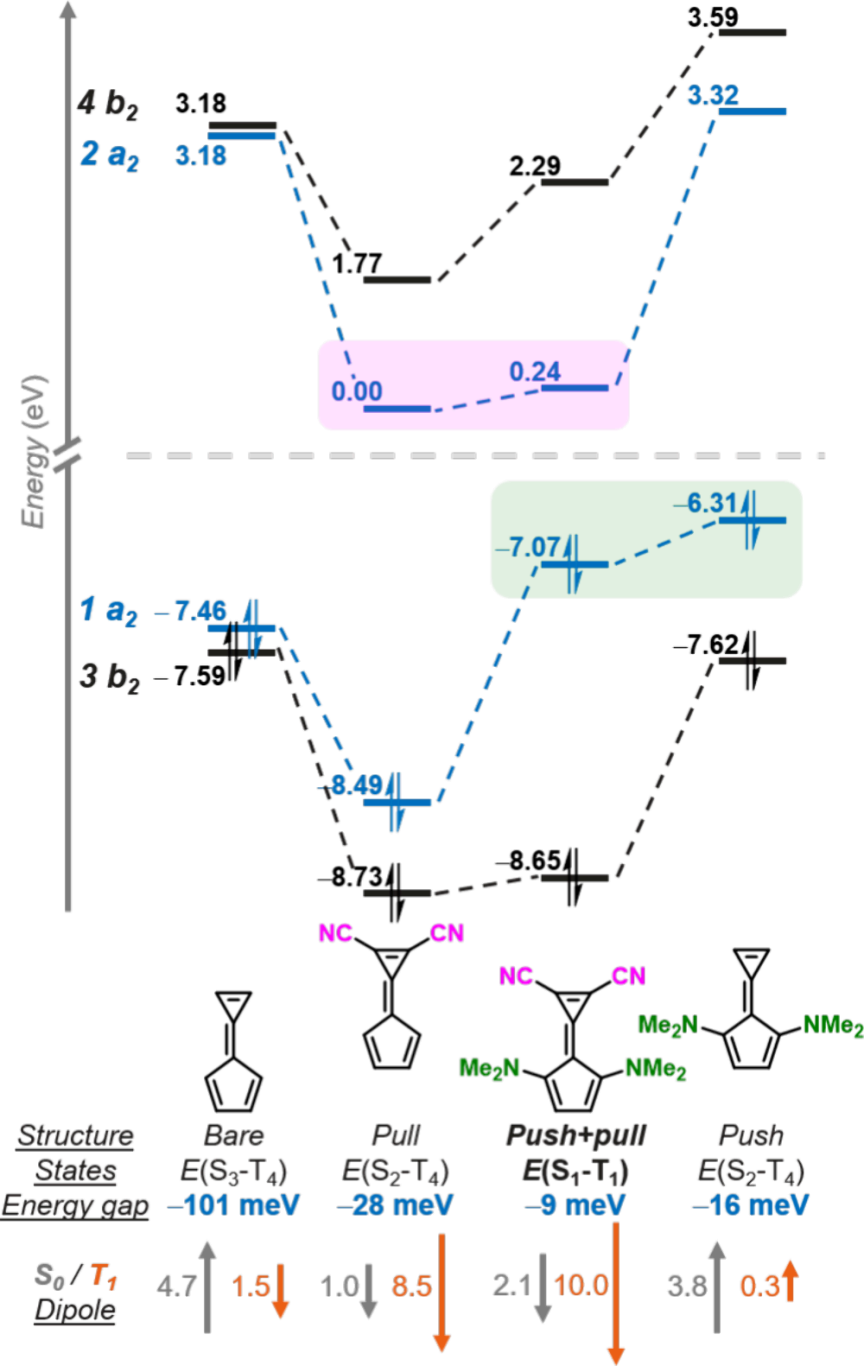
Partial orbital diagram of the two highest occupied and two lowest unoccupied molecular orbitals in calicene and derivatives substituted with electron-donating (*push*) and withdrawing (*pull*) groups. The *a*_2_ orbitals dominating the excitations corresponding to the Hund’s rule violation and the magnitude of the negative gap between these states are shown in blue. Magnitude and direction of the dipoles of the ground state and T_1_ minima are shown in gray and orange, respectively. Energy axis not to scale.

Similarly, a large gap is introduced between the near-degenerate HOMOs by substituting at the 5-membered ring with a strong electron-donating *push* group (−NMe_2_, green in Figure 3) the *a*_2_ HOMO is destabilized by more than 1 eV relative to the bare calicene, and the violation is subsequently lowered from S_3_ to S_2_ with *E*(S_2_–T_4_) = −16 meV. Analogous to the effect of the *pull* substitution above, this CT-type HOMO is more sensitive to the *push* substitution than the other FMOs as it is localized entirely on the 5-membered ring (Figure 2c).

Finally, we apply both *push* (orbital destabilizing) and *pull* (orbital stabilizing) substituent strategies in tandem. This way, the gap between the occupied and unoccupied CT-type *a*_2_ orbitals is reduced considerably relative to the *b*_2_ orbitals, and the resulting *push–pull* calicene becomes a true inverted-gap molecule with an inversion between the S_1_ and T_1_ states with *E*(S_1_–T_1_) = −9 meV; further results in Table S2. Across all four compounds, the NTOs of the states violating Hund’s rule closely resemble the *a*_2_ orbitals shown in Figure 2c (see Figure S1).

Calicene is aromatic in the ground state and, due to its adaptive aromatic nature, exhibits a reversal of its dipole between the ground and lowest excited state.^27,43,44,50^ The *push–pull* substitution pattern proposed here counteracts the dipolar aromatic resonance structure of calicene, leading to a reduction of ground-state aromaticity (Figure S2) and a reduction or reversal in the ground-state dipoles in the substituted compounds (gray in Figure 3). The substituted compounds do not exhibit dipole reversal, and the excited (T_1_) state dipoles of the *pull* and *push–pull* compounds are much larger than in the bare compound (orange in Figure 3). Thus, it can be understood that the CT-type states become the lowest excited states by virtue of the substituents counteracting the intrinsic dipole of the calicene motif. As a result, the ground-state aromaticity of the substituted compounds is attenuated relative to bare calicene. Furthermore, the *push–pull* derivative is mildly Baird aromatic in both rings in the T_1_ state, unlike calicene^27,50^ and the *push* and *pull* derivatives (Figure S2).

We explored functionalization of calicene at various positions and with different substituents to evaluate the scope of the Hund’s rule violation. The *a*_2_ occupied orbital has pronounced features on the carbon atoms of the 5-membered ring adjacent to the exocyclic double-bond (*R*_2_ position in Figure 4), while the *b*_2_ occupied orbital does not (see Figure 2c). By placing electron-donating groups at the *R*_2_ position, the energy of the *a*_2_ HOMO is raised selectively relative to the *b*_2_ orbital. Conversely, donor substitution solely at the *R*_3_ position on the outside of the 5-membered ring destabilizes both occupied orbitals and in fact can eliminate the Hund’s rule violation (e.g., *E*(S_4_–T_4_) = +65 meV for −OH substitution, Figure 4). We attribute this to the delocalized *b*_2_ HOMO also exhibiting orbital coefficients at the *R*_3_ position—in addition to the CT-type *a*_2_ HOMO—thus negating the selective destabilization effect of the *push* strategy. This underlines the sensitivity of Hund’s rule violations to chemical composition, given the very small energy differences between the relevant states. Substituting with donors simultaneously at both available positions on the 5-membered ring preserves the Hund’s rule violation, but changes the order of the state state pair to S_3_-T_4_. Once an acceptor and donor have been placed at the *R*_1_ and *R*_2_ positions, respectively, further functionalization at the *R*_3_ position has no effect on either the magnitude or order of the violation (Figure 4). Reversal of the substituent strategy—by placing electron-donating groups on the 3-membered ring or electron-withdrawing groups on the 5-membered ring—also increases the state order of the Hund’s rule violation or eliminates it entirely (Table S3).

**Figure 4:**
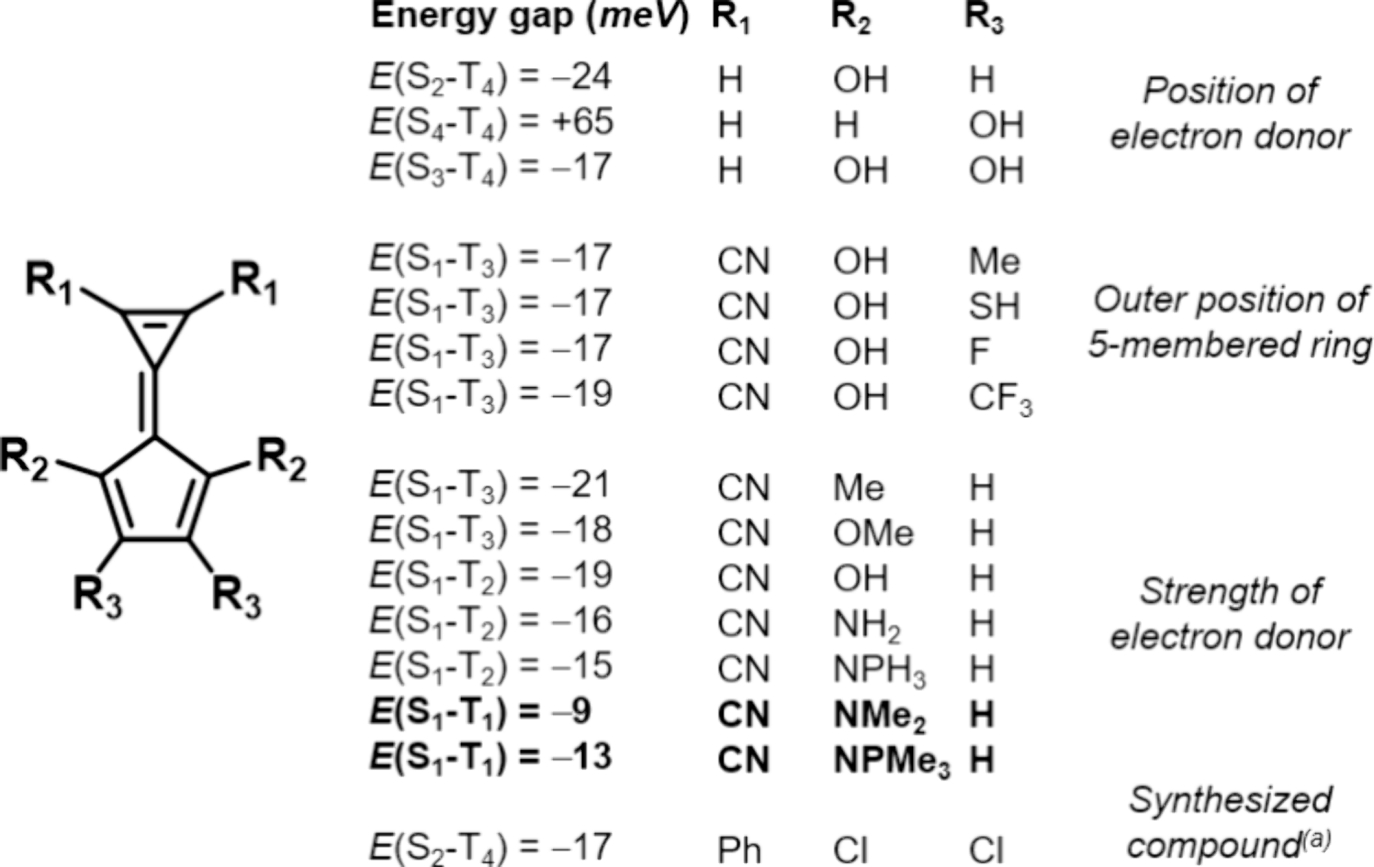
Energy gaps between singlet and triplet excited states in some substituted calicenes. Full results are presented in Table S3. ^*a*^Synthesis reported by Bergmann and Agranat.^51^

A number of electron-withdrawing and -donating substituents were tested at the *R*_1_ and *R*_2_ positions, respectively. We find that the Hund’s rule violations are robust with various substituents (Figure 4 and Table S3), but note that only the strongest donating and withdrawing groups which remain in the plane of the calicene rings are sufficient to maintain the inversion between the S_1_ and T_1_ states at the EOM-CCSD level. However, the specific order of the states in the triplet manifold is method-dependent; with the CC2 method we find that more inversions are potentially between the S_1_ and T_1_ states than the EOM-CCSD computations suggest.

Electron-donating groups weaker than −NMe_2_, such as −OH, −OMe, −NH_2_, −Me, and −NPH_3_ (Figure 3) all exhibit inversions in the S_1_ state when placed at the *R*_2_ position, but the corresponding triplet of same configuration is not the T_1_ state. The only other *R*_2_ substituent we find which contributes to a negative S_1_-T_1_ gap at the EOM-CCSD level when used in conjunction with the −CN acceptor is the extremely strong alkylphosphazene (−NPMe_3_) donor (*E*(S_1_–T_1_) = −13 meV, see Figure S3 for the full state diagram).

A review of the experimental literature reveals that many calicene derivatives have been synthesized, most commonly with alkyl, aryl and halide substituents.^51−54^ We evaluated one synthesized derivative^51^ which is small enough to be computed at the EOM-CCSD level and found that it has a Hund’s rule inversion in the S_2_ state (see Figure 4). While bare calicene has not been synthesized, reports of the synthesis of substituted derivatives containing strong electron-withdrawing groups^55,56^ suggest that *push–pull* compounds such as those reported here may be feasible.

In the NMe_2_-substituted *push–pull* compound discussed in Figure 3, the T_1_-T_3_ states are very close in energy to one another (*E*(T_3_−T_1_) = 0.1 eV, see Figure S3). The energetic proximity of multiple states of different (delocalized and CT) character to the lower S_1_ state may provide efficient channels for reverse intersystem crossing.^4,57^ By contrast, in the more strongly polarized NPMe_3_-substituted compound, the CT-type T_1_ state is 0.25 eV below the next lowest triplet (see Figure S3).

We further examine the excited state energies of these two compounds with S_1_-T_1_ inversions at their excited state minima (S_1_, T_1_, T_2_ and T_3_, see Figures S4–S5). In both compounds, the Hund’s rule violation is preserved across all excited state minima considered. In particular, the Hund’s rule violation persists in the S_1_ and T_1_ states at both the S_1_ and T_1_ minima, i.e., the inversion is still at S_1_-T_1_. It is therefore possible that the singlet–triplet inversion can help optimize the photophysical properties in this class of molecules.

Triafulvene and pentafulvene consist of a three- and five-membered ring, respectively, and an exocyclic double bond (Figure S6). They can be seen as the parent compounds of calicene. Neither of these, nor the donor- and acceptor-substituted derivatives thereof, display a pair of nonoverlapping orbitals. Consequently, none exhibits a Hund’s rule violation in its low-lying excited states, highlighting that the push–pull mechanism inherent to calicene is a necessary requirement to achieve a CT-type inversion.

The next larger push–pull fulvalene is sesquifulvalene, in which the three-membered ring is replaced by a seven-membered ring (see Figure S6 for structure).^58^ Analogous to calicene, the ground state possesses a dipolar resonance structures and aromatic character.^50^ However, despite exhibiting similar orbital features to calicene, no Hund’s rule violation exists among any of its excited states (Figure S7). This may be due to its lower intrinsic push–pull character: the dipole of sesquifulvalene is 3.3 D compared to 4.7 D for calicene.^50^ We applied the strategy described for calicene above, where electron-donating and -withdrawing substituents were placed at the relevant orbital positions to minimize the orbital gap between the CT-type orbitals, but only one compound with a high-lying violation could be located (Table S4) at the EOM-CCSD/cc-pVDZ level.

CT-type inversion of the S_1_ and T_1_ states has been discussed previously in different contexts.^59−61^ Bonačić-Koutecký and Michl have observed that a highly twisted zwitterionic aminoborane (H_2_B-NH_2_, Figure S6)—corresponding to its relaxed S_1_ geometry—yields a negative gap using configuration interaction.^59^ These states are described by an electron transfer mechanism between the occupied nitrogen *p*-orbital lying at a 90° dihedral angle to the unoccupied boron *p*-orbital. The coupled-cluster-based methods we report here predict a small positive gap (50 meV) for aminoborane in its relaxed S_1_ geometry (Table S5). However, with a small structural perturbation (methylation at both B and N), the orthogonal geometry is preserved in the S_1_ state and the gap becomes negative at the EOM-CCSD/cc-pVDZ level (*−*9 meV, Table S5). By contrast, the calicene derivatives reported here are both organic and planar. Unlike CT states formed via couplings between orthogonal donor and acceptor fragments, the states violating Hund’s rule here are not true dark states, but instead exhibit some oscillator strength (Table S2).

In summary, we have identified the first organic molecules to exhibit a negative singlet–triplet gap in a through-bond CT state. By substituting calicene, which exhibits a Hund’s rule violation in upper excited states, the existing inversion is lowered to S_1_ and T_1_, a property which is preserved in the excited state geometries. This occurs by tuning the energies of the orbitals corresponding to these states’ most significant configurations. The functionalization is shown to reverse the dipole and suppress both the ground-state aromaticity and excited state antiaromaticity relative to bare calicene.

This result provides a conceptual link between two pre-existing design principles for organic light-emitting molecules. On one hand, a prevailing strategy for TADF emitters involves minimizing the (positive) singlet–triplet gap by tuning the spatial separation of HOMO and LUMO on distinct and often orthogonal fragments in donor–acceptor-type compounds, leading to the lowest excited states exhibiting through-bond or through-space CT character with significant electron–hole separation. On the other hand, both multiresonance TADF emitters^35−37^ and the emerging family of inverted singlet–triplet gap molecules^1^ rely on patterns of spatially proximate though nonoverlapping frontier MOs, such that the optically relevant states are not charge-separated and instead both the electron and hole are located on the same fragment. The compounds presented here simultaneously exhibit clear CT character emerging from electron–hole separation onto the donor and acceptor fragments of a single conjugated scaffold and a negative singlet–triplet gap without the alternating HOMO/LUMO orbital features. Such a combination of properties may be useful for applications requiring the harvesting of spin-pure electrons from a CT state, such as chirality-induced spin selectivity.^62^

## Supporting Information Available

Computational details, method benchmarking, natural transition orbitals, detailed state diagrams, and full table of results for substituted calicenes at the CC2 and EOM-CCSD levels, and additional data for related molecules (PDF)
XYZ Files (ZIP)
