# Supplementary material for: Singlet–Triplet Inversions in Through-Bond Charge-Transfer States
Source: J Phys Chem Lett. 2024 Sep 26;15(40):10062–7. doi: 10.1021/acs.jpclett.4c02317 (PMC11472380; doi:10.1021/acs.jpclett.4c02317)
Supplement: Supplementary file 1 — jz4c02317_si_001.pdf [file jz4c02317_si_001.pdf]

Supporting Information for "Singlet-Triplet Inversions in Through-Bond Charge-Transfer States"

J. Terence Blaskovits, Clémence Corminboeuf,* and Marc H. Garner*

Laboratory for Computational Molecular Design, Institute of Chemical Sciences and Engineering, École Polytechnique Fédérale de Lausanne (EPFL), 1015 Lausanne, Switzerland

E-mail: clemence.corminboeuf@epfl.ch; marc.garner@epfl.ch

S1. Computational details

All geometry optimizations were performed without symmetry constraints at the ωB97X-D/def2-TZVP [1,2] level using the Gaussian16 package (rev D.01), as were Tamm-Dancoff approximated time-dependent density functional theory computations (TDA-TDDFT). [3] Frequency computations confirmed that all ground-state structures were energy minima with no imaginary frequencies. Approximate second-order coupled-cluster [4] (CC2) and spin-component-scaled [5] CC2 (SCS-CC2) computations were run using the aug-cc-pVDZ basis set [6] with the Turbomole program (v7.1). [7,8] Equations-of-motion coupled cluster with singles and doubles (EOM-CCSD) computations were performed as implemented in the Q-Chem package (v5.1). [9] All orbitals, state energies and isosurfaces provided in the figures were obtained at the EOM-CCSD/cc-pVDZ level. Dipole moments are reported at the ωB97X-D/def2-TZVP level. Excited state character was evaluated via a fragment-based analysis of the TDA-TDDFT transition density matrices using TheoDORE (version 1.7.2). [10] For this, the Gaussian output files were parsed with cclib. [11] in which the percent of charge transfer of a given excited state is obtained from the accumulation of hole and electron density on user-defined molecular fragments. In this instance, we considered the atoms belonging to the 5-membered rings of the calicene derivatives as one fragment, and those belonging to the 3-membered rings as another. Electron-hole separation was evaluated as the distance between the centroids of the electron and hole distributions using Multiwfn. [12] Nucleus-independent chemical shifts were evaluated using the out-of-plane components of the magnetic shielding tensor at 1 Å above the centroid of each ring (NICS$(1)_{zz}$) [13] at the B3LYP/6-31G(d) level [14,15] using the gauge-independent atomic orbital method. [16,17]

S2. Choice of methods

Benchmarking of calicene with the aug-cc-pVDZ, cc-pVTZ and aug-cc-pVTZ basis sets reveals very good agreement in state ordering and the magnitude of the Hund´s rule inversion (Table S1), in agreement with our previous observation of the small basis set dependence in

(quasi-)planar conjugated molecules.[18] As such, we have used the cc-pVDZ basis set for the all remaining computations to ensure tractability for larger molecules.

Although no experimental data is available for unsubstituted calicene (which has not been isolated), the measured $E(\mathrm{S}_1{-}\mathrm{T}_1)$ in azulene (49 meV)[19] is only slightly lower than the EOM-CCSD/cc-pVDZ-computed value (55 meV).[?] This indicates that EOM-CCSD/cc-pVDZ is an appropriate method for evaluating aromatic compounds with zwitterionic character, and highlights that this method even slightly overestimates singlet-triplet gaps, thus providing a conservative assessment of negative singlet-triplet gaps in conjugated molecules.[18]

Finally, we note that approximate second-order coupled cluster (CC2)[4] systematically shifts the Hund´s rule inversion to lower excited states (Table S1), in keeping with our previous observation for zwitterionic compounds.[20] For instance, at the CC2/aug-cc-pVDZ level, the inversion in *bare* calicene is predicted to occur in the S_2-T_4 state pair (rather than in S_3-T_4 obtained with EOM-CCSD), and in S_1-T_2 in the *pull* derivative rather than in S_2-T_4 (EOM-CCSD/cc-pVDZ).

S3. Additional figures and tables

All optimized structures are available as supporting files in *xyz* format. Compound names corresponding to the *xyz* coordinate files are provided in the relevant tables.

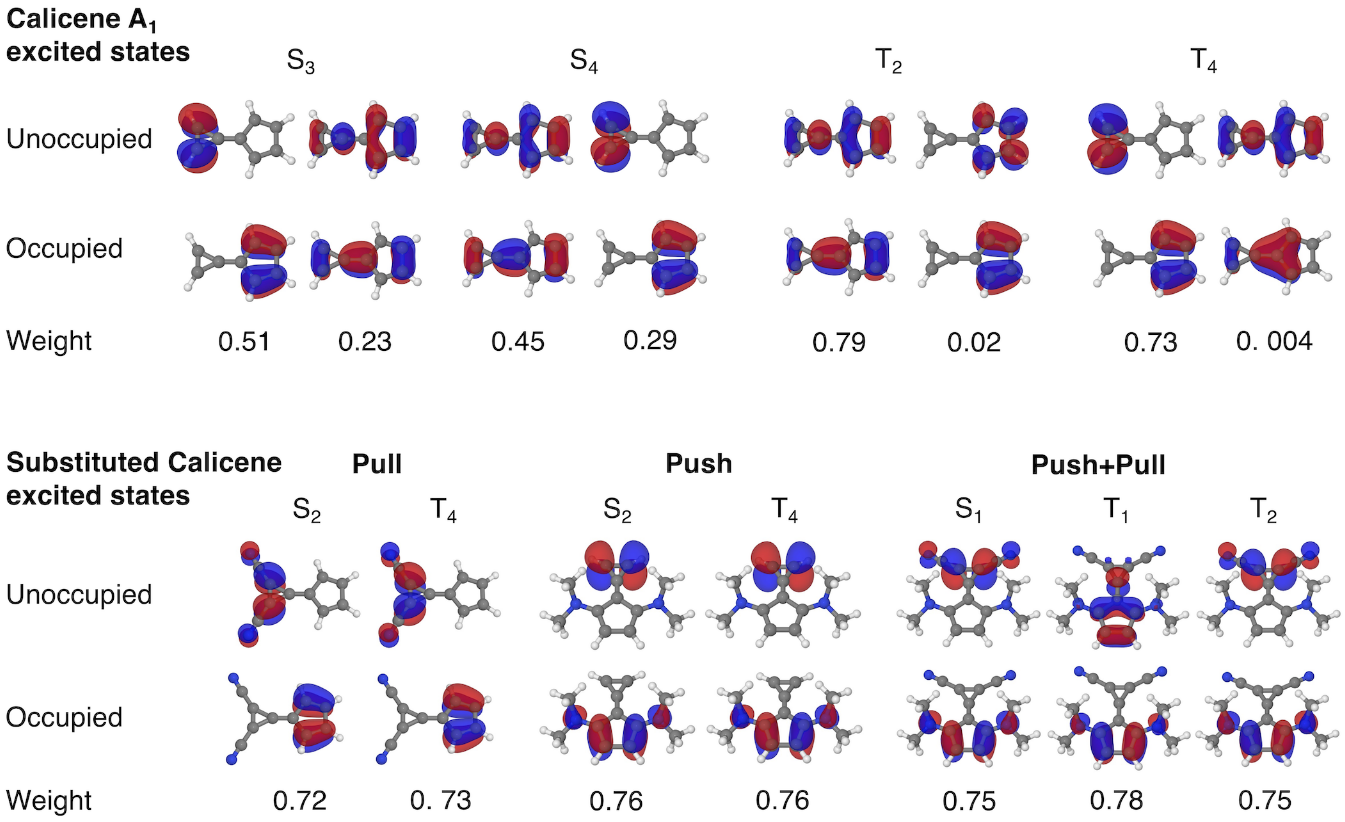

Figure S1: Natural transition orbital (NTO) pairs for the A_1 excited states in bare calicene (top) and the excited states violating Hund's rule in *pull*-, *push*- and *push-pull*-substituted derivatives (below), evaluated at the EOM-CCSD/cc-pVDZ//ωB97X-D/def2-TZVP level. The two most important NTO pairs are shown for the calicene excited states, while only the most important pair is shown in the substituted systems.

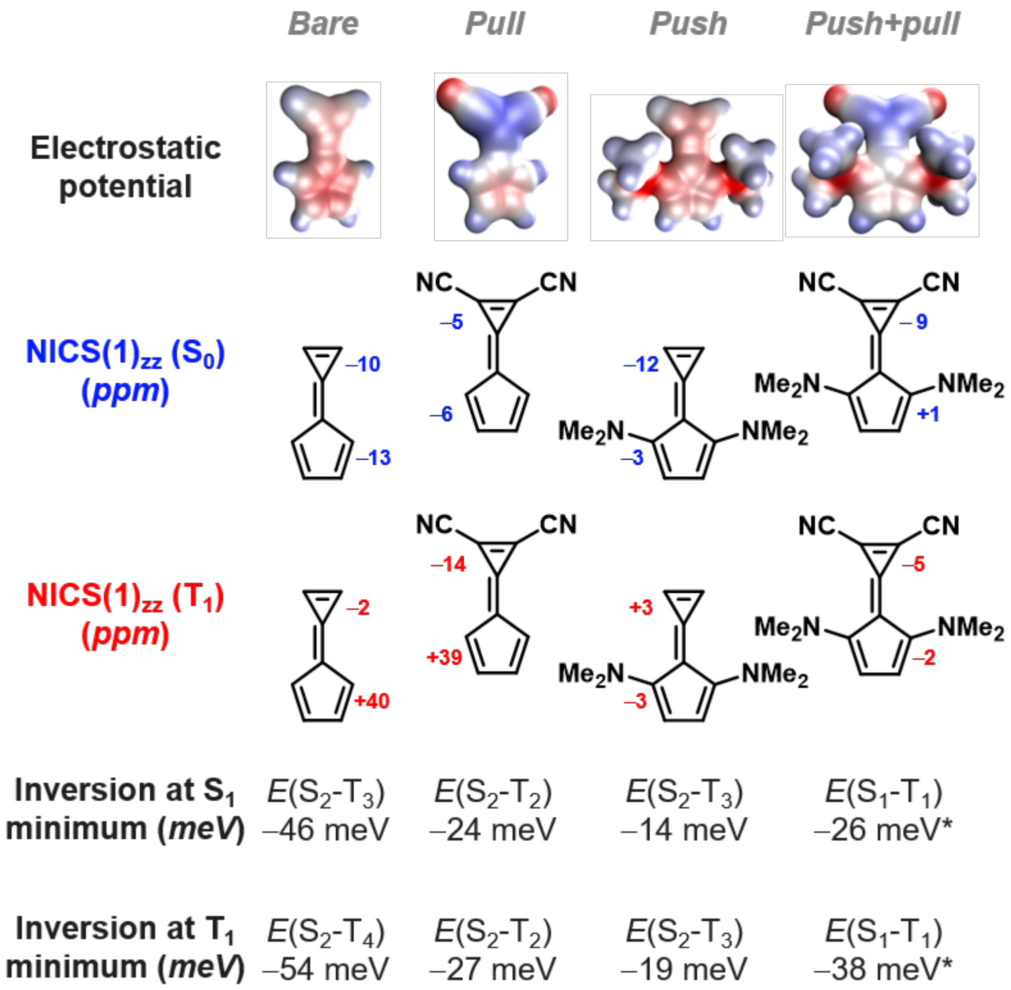

Figure S2: Electrostatic potentials of bare calicene and representative *pull*- *push*- and *push-pull*-substituted derivatives, obtained at the EOM-CCSD/cc-pVDZ//ωB97X-D/def2-TZVP level using a isovalue of 0.1 a.u. and a color range of +/- 0.08. Out-of-plane nucleus-independent chemical shifts (NICS$(1)_{z}z$) in the S_0 ground state (blue) and relaxed T_1 state (red).

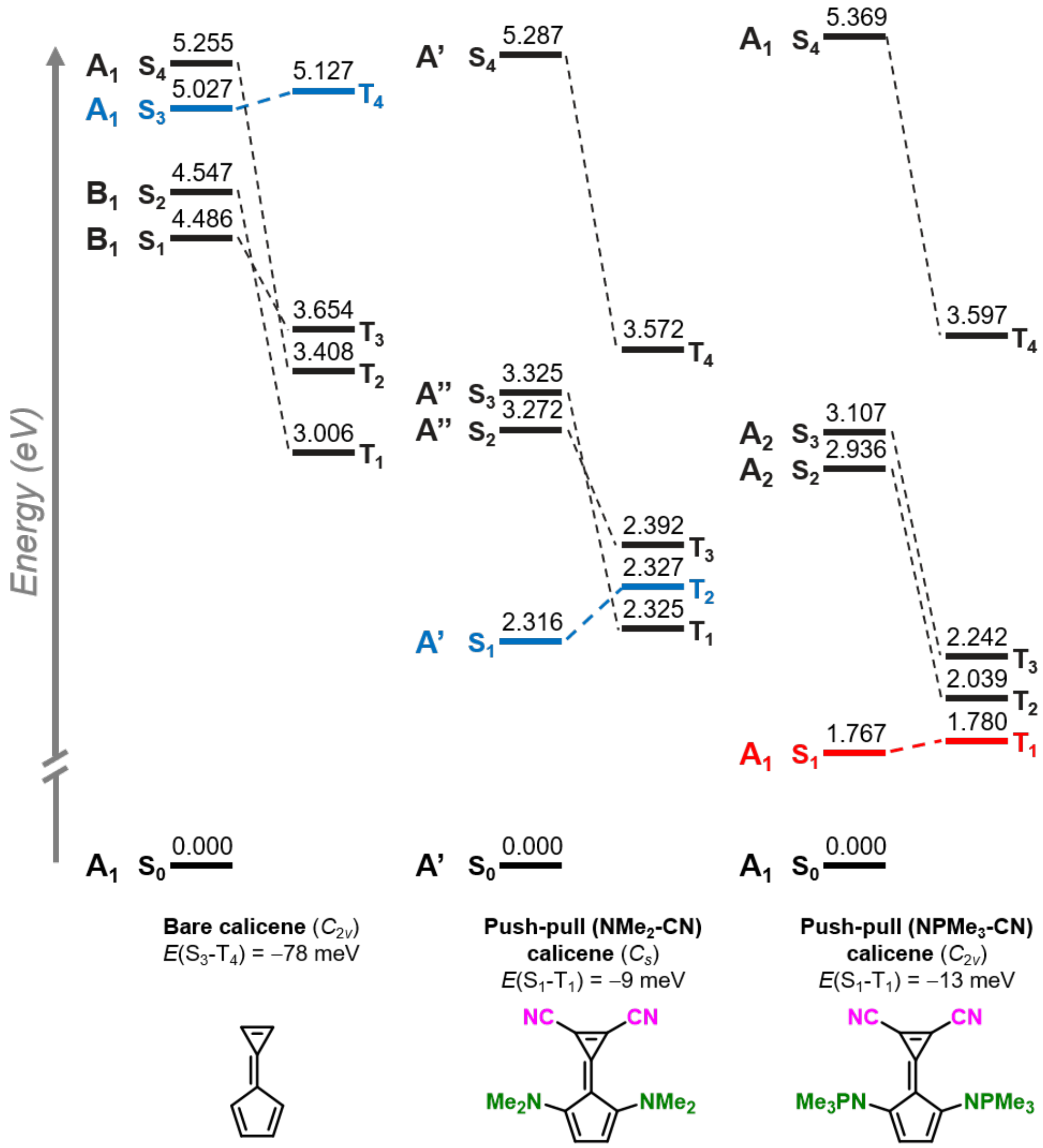

Figure S3: Excited state ordering, energies and symmetries in bare calicene and two push-pull substituted derivatives at their ground state geometries. States corresponding to the Hund's rule inversion are colored. Energies obtained at the EOM-CCSD/cc-pVDZ//ωB97X-D/def2-TZVP level. Energy axis not to scale.

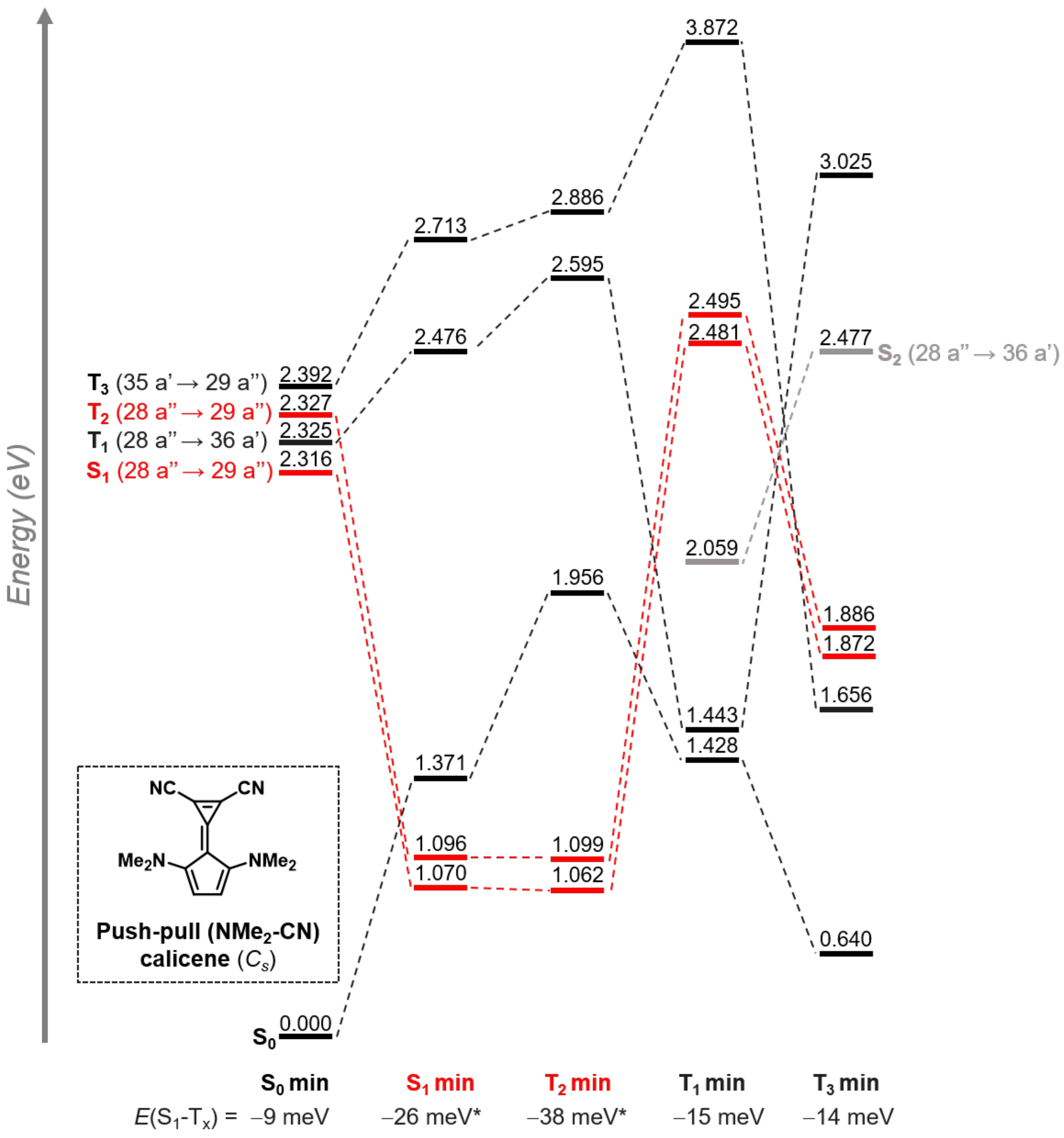

Figure S4: Excited state energies and symmetries of the dimethylamino/nitrile-substituted push-pull calicene at its ground state and relaxed excited state geometries. States corresponding to the Hund's rule inversion are colored. Energies obtained at the EOM-CCSD/cc-pVDZ. Excited state geometries obtained by adiabatic relaxation with TDA-TDDFT at the same level as the ground state optimization (ωB97X-D/def2-TZVP) with symmetry maintained. Energy axis not to scale. * = The states exhibiting the Hund's rule violation are lower in energy than the closed shell configuration solution.

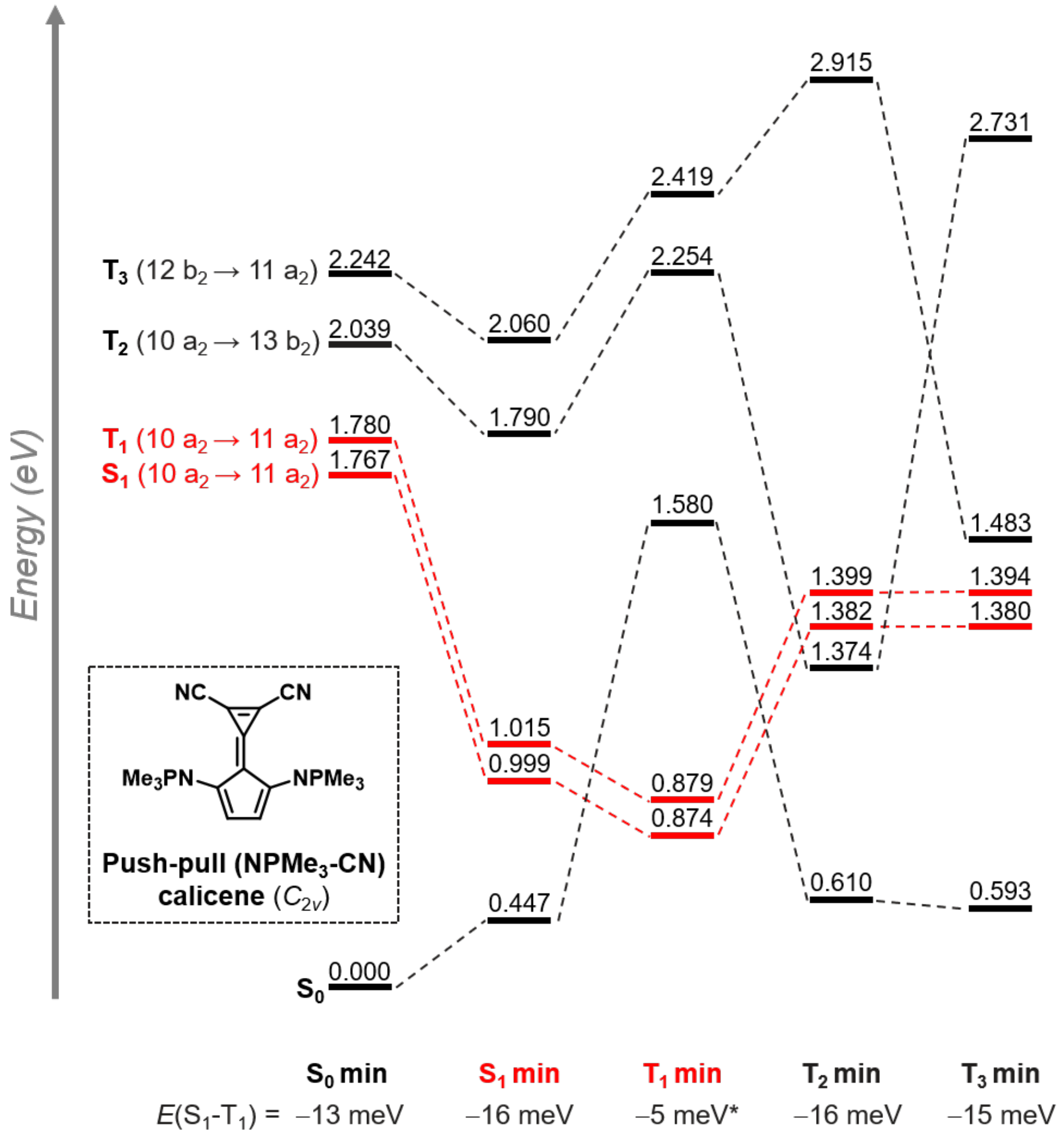

Figure S5: Excited state energies and symmetries of the trimethylphosphazeno/nitrile-substituted push-pull calicene at its ground state and relaxed excited state geometries. States corresponding to the Hund's rule inversion are colored. Energies obtained at the EOM-CCSD/cc-pVDZ. Excited state geometries obtained by adiabatic relaxation with TDA-TDDFT at the same level as the ground state optimization (ωB97X-D/def2-TZVP) with symmetry maintained. Energy axis not to scale. * = The states exhibiting the Hund's rule violation are lower in energy than the closed shell configuration solution.

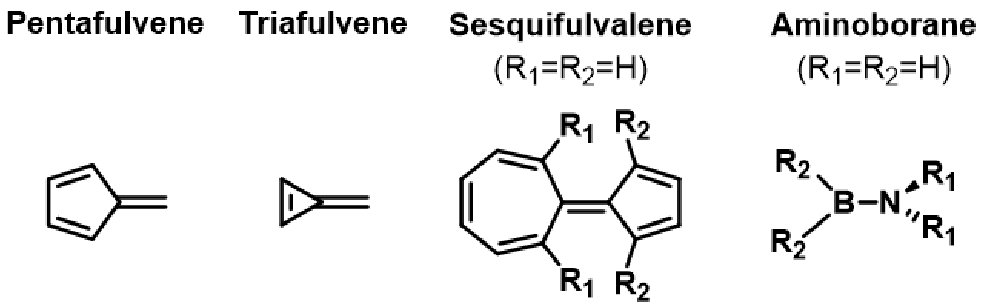

Figure S6: Supplementary compounds discussed in the main text.

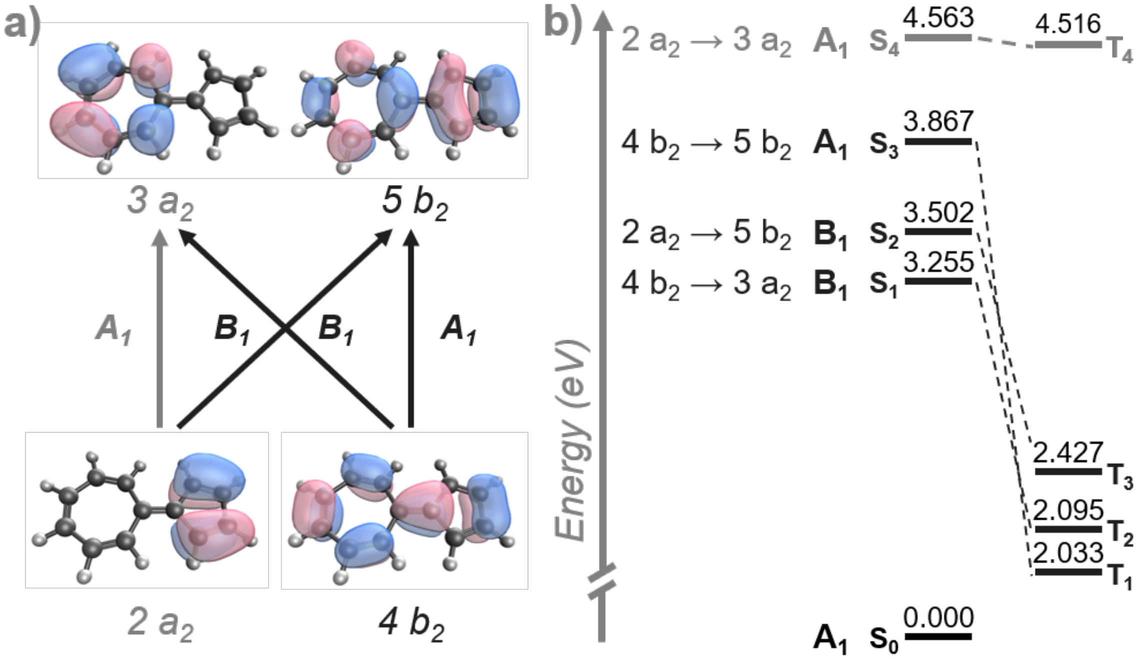

Figure S7: (a) Key orbitals contributing to the lowest excited states in sesquifulvalene; (b) state diagram of sesquifulvalene. The charge-transfer excitation is indicated in grey; it does not violate Hund's rule. Evaluated at the EOM-CCSD/cc-pVDZ//ωB97X-D/def2-TZVP level. Energy axis not to scale.

Table 1: Singlet-triplet gaps of bare calicene and representative *pull*-, *push*- and *push-pull*-substituted derivatives computed using different methods. Energies are given in eV. S_x and T_y correspond to the singlet and triplet states of the same electron configuration exhibiting the Hund's rule inversion. The values of x and y are provided. % Sx (% Ty) is the contribution (in %) of the charge-transfer ($a_2 \rightarrow a_2$) electron configuration to the total S_x (T_y) excitation. Geometries obtained at the ωB97X-D/def2-TZVP level.

MOLECULE	Method	Sx	Ty	E(Sx)	E(Ty)	E(Sx-Ty)	% Sx	% Ty
**bare**	CC2/cc-pVDZ	3	4	4.425	4.450	-0.025	98	91
**(calicene)**	CC2/aug-cc-pVDZ	2	4	4.111	4.138	-0.026	83	84
	SCS-CC2/cc-pVDZ	3	4	5.015	5.084	-0.069	76	98
	EOM-CCSD/cc-pVDZ	3	4	5.027	5.127	-0.101	57	84
	EOM-CCSD/aug-cc-pVDZ	3	4	4.662	4.760	-0.098	54	75
	EOM-CCSD/cc-pVTZ	3	4	4.903	5.016	-0.113	52	83
	EOM-CCSD/aug-cc-pVTZ	3	4	4.701	4.809	-0.108	43	65
**pull**	CC2/cc-pVDZ	1	3	2.873	2.889	-0.016	98	98
**(3ring_cn)**	CC2/aug-cc-pVDZ	1	2	2.764	2.779	-0.015	98	98
	SCS-CC2/cc-pVDZ	2	4	3.533	3.559	-0.016	98	95
	EOM-CCSD/cc-pVDZ	2	4	3.701	3.728	-0.028	85	85
	EOM-CCSD/aug-cc-pVDZ	2	4	3.603	3.627	-0.024	85	85
**push**	CC2/cc-pVDZ	1	2	3.271	3.279	-0.009	98	98
**(5ring_nme2)**	CC2/aug-cc-pVDZ	2	3	3.476	3.415	0.061	53	61
	SCS-CC2/cc-pVDZ	2	4	3.941	3.954	-0.013	98	95
	EOM-CCSD/cc-pVDZ	2	4	4.039	4.055	-0.016	85	85
	EOM-CCSD/aug-cc-pVDZ	2	3	3.749	3.755	-0.005	23	23
**push-pull**	CC2/cc-pVDZ	1	1	1.400	1.409	-0.009	97	97
**(5ring_nme2_3ring_cn_in)**	CC2/aug-cc-pVDZ	1	1	1.310	1.318	-0.008	97	97
	SCS-CC2/cc-pVDZ	1	1	2.095	2.106	-0.011	97	97
	SCS-CC2/aug-cc-pVDZ	1	1	2.005	2.016	-0.011	97	97
	EOM-CCSD/cc-pVDZ	1	1	2.316	2.327	-0.011	85	85

Table 2: Selected properties of bare calicene, representative ***pull***-, ***push***- and ***push-pull***-substituted derivatives, and ***trans***-bicalicene, evaluated at the ωB97X-D/def2-TZVP level. Positive dipole values indicate a vector pointing from the 5-membered to the 3-membered ring, while negative values indicate the opposite. Charge transfer character from the 5-membered to the 3-membered ring was evaluated via a fragment-based analysis of the transision density matrix, as outlined in section S1.

NAME	bare (calicene)	pull (calicene_3ring_cn)	push (5ring_nme2)	push-pull (5ring_nme2_3ring_cn_in)	Trans-bicalicene
Symmetry point group	*C2v*	*C2v*	*Cs*	*Cs*	*D2h*
Sx state label	1A1 (S3)	1A1 (S2)	1A' (S2)	1A' (S1)	1B1g (S1)
Ty state label	2A1 (T4)	2A1 (T4)	2A' (T4)	1A' (T2)	1B1g (T2)
S0 Dipole (*Debye*)	4.7	-1.0	3.8	-2.1	0.0
T1 Dipole (*Debye*)	-1.5	-8.5	0.3	-10.0	4.3
Sx osc (*a.u.*)	0.0094	0.0008	0.0008	0.0017	0.0000
Sx 5-membered to 3-membered CT (*%*)	95	99	96	94	37
Sx e-h separation (*A*)	3.61	4.25	3.62	4.01	0.00
Tx e-h separation (*A*)	3.69	4.14	3.61	4.01	0.00

Table S3: Singlet-triplet energies and gaps of calicene derivatives computed at the CC2/aug-cc-pVDZ and EOM-CCSD/cc-pVDZ levels. S_x and T_y correspond to the singlet and triplet states of the same electron configuration exhibiting the Hund's rule inversion. The values of x and y are provided. Energies are given in eV. Geometries obtained at the ωB97X-D/def2-TZVP level. DNC = did not converge at the level of theory indicated. * = For compounds where no Hund's rule inversion was located at the CC2 level, the S_1-T_1 gap is provided.

				CC2					EOM-CCSD				
**NAME**	**R1**	**R2**	**R3**	**Sx**	**Ty**	**E(Sx)**	**E(Ty)**	**E(Sx-Ty)**	**Sx**	**Ty**	**E(Sx)**	**E(Ty)**	**E(Sx-Ty)**
**calicene**	H	H	H	2	4	4.111	4.138	-0.026	3	4	5.027	5.127	-0.101
**BH2**	BH2	H	H	4	4	3.249	3.252	-0.003					
**3ring_cn**	CN	H	H	1	2	2.764	2.779	-0.015	2	4	3.701	3.728	-0.028
**5ring_nh2_in**	H	NH2	H	1	2	3.003	3.006	-0.003	2	4	4.031	4.051	-0.020
**5ring_nh2_out**	H	H	NH2	2	4	3.889	3.913	-0.024					
**5ring_nh2_both**	H	NH2	NH2	2	5	3.585	3.594	-0.010					
**5ring_oh_in**	H	OH	H	1	2	3.415	3.423	-0.007	2	4	4.412	4.436	-0.024
**5ring_oh_out***	H	H	OH	1	1	3.716	3.178	0.537	4	4	5.305	5.240	0.065
**5ring_oh_both**	H	OH	OH	2	4	3.398	3.403	-0.005	3	4	4.438	4.454	-0.017
**3ring_nh2***	NH2	H	H	1	1	3.377	3.344	0.033					
**3ring_oh***	OH	H	H	1	1	3.399	3.214	0.186					
**5ring_bh2_in***	H	BH2	H	1	1	3.483	2.624	0.859					
**5ring_bh2_out***	H	H	BH2	1	1	3.768	2.916	0.852					
**5ring_bh2_both***	H	BH2	BH2	1	1	3.420	2.664	0.756					
**5ring_cn_in***	H	CN	H	1	1	4.045	2.881	1.164	4	5	5.3124	5.4085	-0.096
**5ring_cn_out**	H	H	CN	3	4	4.332	4.383	-0.052	4	5	5.3291	5.3765	-0.047
**5ring_cn_both**	H	CN	CN	3	5	4.663	4.744	-0.081	4	6	5.4348	5.4839	-0.049
**5ring_oh_3ring_bh2_in***	BH2	OH	H	1	1	2.582	2.574	0.008					
**5ring_oh_3ring_cn_in**	CN	OH	H			DNC			1	2	2.819	2.838	-0.019
**5ring_oh_3ring_cn_out**	CN	H	OH	2	2	2.685	2.698	-0.012					
**5ring_oh_3ring_cn_both**	CN	OH	OH	1	1	1.855	1.864	-0.010					
**5ring_nh2_3ring_cn_in**	CN	NH2	H	1	1	1.477	1.486	-0.009	1	2	2.393	2.409	-0.016
**5ring_nh2_3ring_cn_out**	CN	H	NH2	2	2	2.406	2.418	-0.013					
**5ring_nh2_3ring_cn_both***	CN	NH2	NH2	1	1	1.342	1.259	0.084					
**5ring_me_3ring_cn_in**	CN	Me	H	1	1	2.228	2.239	-0.011	1	3	3.249	3.270	-0.021
**5ring_me_3ring_cn_out**	CN	H	Me	1	2	2.635	2.649	-0.014					
**5ring_me_3ring_cn_both**	CN	Me	Me	1	1	2.102	2.111	-0.010	2	3	3.182	3.200	-0.018
**5ring_me_3ring_cf3_in***	CF3	Me	H	1	1	3.775	2.732	1.043	3	4	4.199	4.224	-0.025
**5ring_ome_3ring_cn_in**	CN	OMe	H	1	1	1.773	1.783	-0.010	1	3	2.768	2.785	-0.018
**5ring_ome_3ring_cn_out**	CN	H	OMe	2	2	2.632	2.644	-0.012	2	4	3.654	3.667	-0.013
**5ring_nme2_3ring_cn_in**	CN	NMe2	H	1	1	1.310	1.318	-0.008	1	1	2.316	2.327	-0.011
**5ring_sh_3ring_cn_in**	CN	SH		1	2	2.550	2.561	-0.011	2	3	3.544	3.565	-0.021
**me_oh_cn**	CN	OH	Me	1	1	1.746	1.756	-0.010	1	3	2.740	2.757	-0.017
**sh_oh_cn**	CN	OH	SH	1	1	1.846	1.857	-0.011	1	3	2.843	2.860	-0.017
**f_oh_cn**	CN	OH	F	1	1	2.048	2.058	-0.010	1	3	2.972	2.990	-0.017
**cf3_oh_cn**	CN	OH	CF3	1	1	2.310	2.322	-0.012	1	3	3.245	3.264	-0.019
**5ring_nh2_3ring_NO2**	NO2	NH2	H	1	1	1.099	1.107	-0.008	1	2	2.078	2.087	-0.009
**5ring_nph3_3ring_CN**	CN	NPH3	H	1	1	1.255	1.264	-0.009	1	2	2.195	2.210	-0.015
**5ring_npme3_3ring_CN**	CN	NPMe3	H	1	1	0.792	0.802	-0.010	1	1	1.767	1.780	-0.013
**5ring_nme2_3ring_nn+**	NN+	NMe2	H	1	2	2.434	2.458	-0.025	3	5	3.238	3.252	-0.014
**5ring_nhme_3ring_nme3+**	NMe3+	NHMe	H			DNC			1	2	3.101	3.108	-0.007
**5ring_nme2**	H	NMe2	H			DNC			2	4	4.039	4.055	-0.016
**5ring_npme3**	H	NPMe3	H			DNC			2	4	3.737	3.766	-0.028
**5ring_oh_3ring_cf3**	CF3	OH	H	1	4	4.004	3.958	0.046	2	4	3.812	3.836	-0.024
**5ring_nph3_3ring_NO**	NO	NPH3	H	1	2	1.040	0.969	0.071	4	6	3.000	3.257	-0.257
**5ring_nh2_3ring_NO***	NO	NH2	H	1	1	3.020	1.900	1.120	8	11	5.222	5.237	-0.015
**CLOFUL**	Ph	Cl	Cl	2	3	2.941	2.952	-0.011	2	4	3.995	4.012	-0.017

Table S4: Singlet-triplet energies and gaps of sesquifulvalene derivatives evaluated at the EOM-CCSD/cc-pVDZ//ωB97X-D/def2-TZVP level. S_x and T_y correspond to the singlet and triplet states of the same electron configuration exhibiting the Hund's rule inversion. The values of x and y are provided. The structure of sesquifulvalene and the positions of the R substituents are shown in Figure S6. Energies are given in eV.

**name**	**R1 (7-mem)**	**R2 (5-mem)**	**Sx**	**Ty**	**E(Sx)**	**E(Ty)**	**E(Sx-Ty)**
**sesquifulvalene**	H	H	4	5	4.5631	4.5163	0.047
**sesqui_5ring_oh**	H	OH	3	4	4.0162	4.0391	-0.023
**sesqui_5ring_oh_7ring_2f_in**	F	OH	4	8	5.3912	5.3917	-0.001
**sesqui_5ring_oh_7ring_2cn_in**	CN	OH	2	5	4.0235	4.0206	0.003
**sesqui_7ring_2cn_in**	CN	H	4	5	4.7313	4.7005	0.031
**sesqui_5ring_2nh2**	H	NH2	2	4	3.9305	3.9244	0.006
**sesqui_7ring_2NN+**	NN+	H	2	4	3.1129	3.1093	0.004
**sesqui_5ring_2nph3**	H	NPH3	2	4	3.701	3.6984	0.003

Table S5: Singlet-triplet energies and gaps of aminoborane derivatives evaluated at the EOM-CCSD/cc-pVDZ//ωB97X-D/def2-TZVP level on the S_1 minimum geometry. The structure of aminoborane and the positions of the R substituents are shown in Figure S6. Energies are given in eV.

NAME	R1	R2	CC2 E(S1)	E(T1)	E(S1-T1)	EOM-CCSD E(S1)	E(T1)	E(S1-T1)
aminoborane	H	H	3.099	3.029	0.070	3.090	3.043	0.047
N,N-dimethylaminoborane	CH3	H	2.059	2.036	0.023	2.172	2.158	0.014
tetramethylaminoborane	CH3	CH3	2.738	2.748	-0.010	2.919	2.928	-0.009

References

(1) J.-D. Chai, M. Head-Gordon Long-range corrected hybrid density functionals with damped atom–atom dispersion corrections. *Phys. Chem. Chem. Phys.* **2008**, *10*, 6615–6620.

(2) F. Weigend, R. Ahlrichs Balanced basis sets of split valence, triple zeta valence and quadruple zeta valence quality for H to Rn: Design and assessment of accuracy. *Phys. Chem. Chem. Phys.* **2005**, *7*, 3297.

(3) M. J. Frisch, G. W. Trucks, H. B. Schlegel, G. E. Scuseria, M. A. Robb, J. R. Cheeseman, G. Scalmani, V. Barone, G. A. Petersson, H. Nakatsuji et al. Gaussian 16, Revision A.03. 2016, Gaussian Inc.: Wallingford, CT.

(4) O. Christiansen, H. Koch, P. Jørgensen The second-order approximate coupled cluster singles and doubles model CC2. *Chem. Phys. Lett.* **1995**, *243*, 409–418.

(5) A. Hellweg, S. A. Grün, C. Hättig Benchmarking the performance of spin-component scaled CC2 in ground and electronically excited states. *Phys. Chem. Chem. Phys.* **2008**, *10*, 4119–4127.

(6) T. H. Dunning Gaussian basis sets for use in correlated molecular calculations. I. The atoms boron through neon and hydrogen. *J. Chem. Phys.* **1989**, *90*, 1007–1023.

(7) R. Ahlrichs, M. Bär, M. Häser, H. Horn, C. Kölmel Electronic structure calculations on workstation computers: The program system turbomole. *Chem. Phys. Lett.* **1989**, *162*, 165–169.

(8) TURBOMOLE V7.4 2019, a development of University of Karlsruhe and Forschungszentrum Karlsruhe GmbH, 1989-2007, TURBOMOLE GmbH, since 2007; available from `http://www.turbomole.com`.

(9) E. Epifanovsky, A. T. Gilbert, X. Feng, J. Lee, Y. Mao, N. Mardirossian, P. Pokhilko, A. F. White, M. P. Coons, A. L. Dempwolff et al. Software for the frontiers of quantum chemistry: An overview of developments in the Q-Chem 5 package. *J. Chem. Phys.* **2021**, *155*, 084801.

(10) F. Plasser TheoDORE: A toolbox for a detailed and automated analysis of electronic excited state computations. *J. Chem. Phys.* **2020**, *152*, 084108.

(11) N. M. O'boyle, A. L. Tenderholt, K. M. Langner cclib: A library for package-independent computational chemistry algorithms. *J. Comput. Chem.* **2008**, *29*, 839–845.

(12) T. Lu, F. Chen Multiwfn: A multifunctional wavefunction analyzer. *J. Comput. Chem.* **2012**, *33*, 580–592.

(13) P. v. R. Schleyer, C. Maerker, A. Dransfeld, H. Jiao, N. J. van Eikema Hommes Nucleus-Independent Chemical Shifts: A Simple and Efficient Aromaticity Probe. *J. Am. Chem. Soc.* **1996**, *118*, 6317–6318.

(14) C. Lee, W. Yang, R. G. Parr Development of the Colle-Salvetti correlation-energy formula into a functional of the electron density. *Phys. Rev. B* **1988**, *37*, 785.

(15) A. D. Becke Density-functional thermochemistry. III. The role of exact exchange. *J. Chem. Phys.* **1993**, *98*, 5648–5652.

(16) R. Ditchfield Self-consistent perturbation theory of diamagnetism: I. A gauge-invariant LCAO method for NMR chemical shifts. *Mol. Phys.* **1974**, *27*, 789–807.

(17) K. Wolinski, J. F. Hinton, P. Pulay Efficient implementation of the gauge-independent atomic orbital method for NMR chemical shift calculations. *J. Am. Chem. Soc.* **1990**, *112*, 8251–8260.

(18) M. H. Garner, J. T. Blaskovits, C. Corminboeuf Double-bond delocalization in non-alternant hydrocarbons induces inverted singlet–triplet gaps. *Chem. Sci.* **2023**, *14*, 10458–10466.

(19) S. Vosskötter, P. Konieczny, C. M. Marian, R. Weinkauf Towards an understanding of the singlet–triplet splittings in conjugated hydrocarbons: azulene investigated by anion photoelectron spectroscopy and theoretical calculations. *Phys. Chem. Chem. Phys.* **2015**, *17*, 23573–23581.

(20) J. T. Blaskovits, M. H. Garner, C. Corminboeuf Symmetry-Induced Singlet-Triplet Inversions in Non-Alternant Hydrocarbons. *Angew. Chem. Int. Ed.* **2023**, *62*, e202218156.
